# Gingival metastasis of a mediastinal pulmonary sarcomatoid carcinoma: a case report

**DOI:** 10.1186/s13019-019-0991-y

**Published:** 2019-09-09

**Authors:** Zhonghua Qin, Bin Huang, Guiping Yu, Yongqiang Zheng, Ke Zhao

**Affiliations:** 1 Department of Cardiothoracic Surgery, The affiliated Jiangyin People’s Hospital of Southeast University Medical College, 3 Yingrui Road, Jiangyin, 214400 Jiangsu China; 2 Department of Radiology, The affiliated Jiangyin People’s Hospital of Southeast University Medical College, 3 Yingrui Road, Jiangyin, 214400 Jiangsu China; 3 Department of Pathology, The affiliated Jiangyin People’s Hospital of Southeast University Medical College, 3 Yingrui Road, Jiangyin, 214400 Jiangsu China

**Keywords:** Pulmonary sarcomatoid carcinoma, Non-small cell lung cancer, Gingival tumor metastasis, Video-assisted thoracic surgery

## Abstract

**Background:**

Pulmonary sarcomatoid carcinoma (PSC) is a rare malignancy with both epithelial and sarcoma components, and high tumor metastasis potential.

**Case presentation:**

A 63-year-old male patient had a tumor in the right posterior mediastinum, and was eventually diagnosed with PSC and gingival metastasis. The patient underwent thoracoscopic right upper pneumonectomy with lymph node dissections, and the subsequent gingival biopsy revealed a metastatic PSC. The immunohistochemistry revealed that both PSC site tissues were positive for vimentin, CKAE1/AE3 and Ki-67. The patient received radiotherapy and chemotherapy after surgery, and deceased two months later due to systemic tumor metastases.

**Conclusion:**

PSC metastasis is variable, and leads to diagnostic dilemma or erroneous diagnosis. A differential diagnosis can help to distinguish it from gingival cancer.

## Background

Pulmonary sarcomatoid carcinoma (PSC) is a very rare but aggressive type of non-small cell lung cancer (NSCLC). Clinically, PSC is characterized by tumor cells with histological, cytological, or molecular properties of both epithelial and mesenchymal tumors [1, 2]. PSC could be presented to have a large size with invasive tendency, early recurrence and systemic metastases. The incidence PSC has been reported to range between 0.1 and 0.4% in all diagnosed lung cancers [3]. In the present case report, the patient has a tumor in the right upper lobe of the lung, and suffered from swollen gums. During the hospitalization, the patient underwent surgery, and was histologically diagnosed with PSC, while the gingival biopsy revealed a metastatic PSC. Therefore, the present case is unique and worth reporting.

## Case presentation

**Fig. 1:**
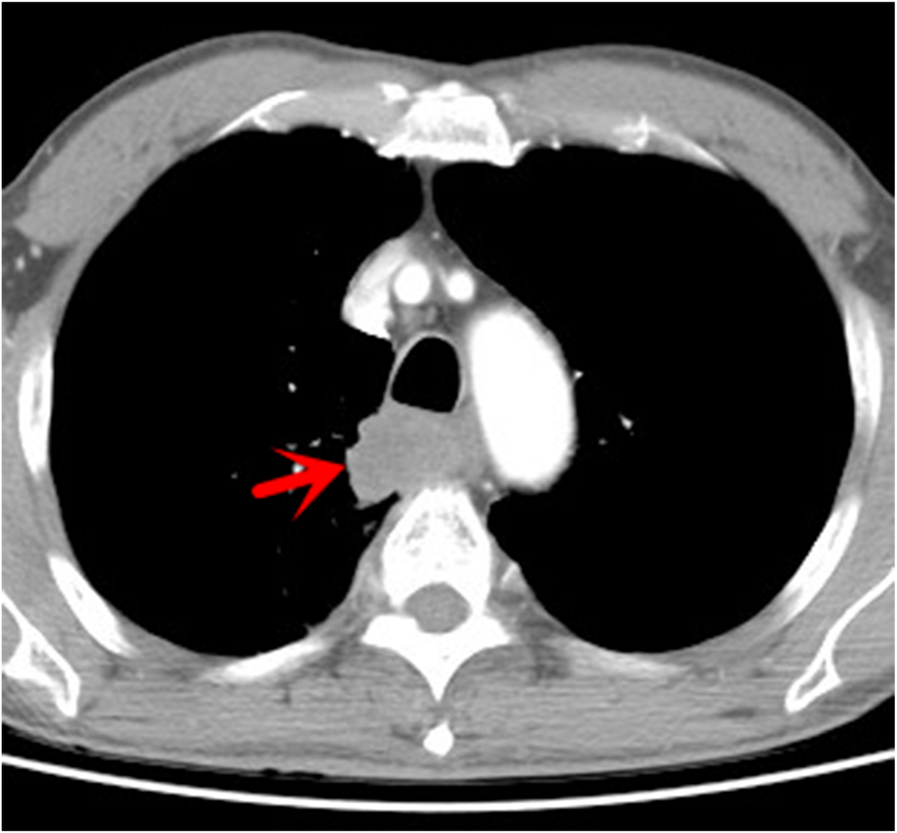
Chest CT images before surgery. The enhanced chest CT revealed a 2.8 × 2.5 cm mass in the right posterior mediastinum (red arrow)

**Fig. 2:**
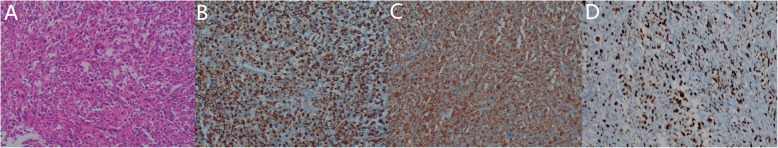
The histology and immunohistochemistry of the lung tumor. **a** Histology. The hematoxylin and eosin (H&E) stained lung tumor tissue shows that the tumor cells contain abundant cytoplasm, erythematous and heteromorphic (× 100). **b** Immunohistochemistry. The data shows the partial positivity of the CKAE1/AE3 immunostaining in tumor cells. **c** Immunohistochemistry. The data shows the partial positivity of the vimentin immunostaining in tumor cells. **d** Immunohistochemistry. The data shows the partial positivity of the Ki-67 immunostaining in tumor cells

A 63-year-old patient with tobacco smoking history had cough and bloodstained sputum for a month. The computed tomography (CT) scan revealed an abnormal mass in the right posterior mediastinum. Subsequently, the patient was admitted to Jiangyin People’s Hospital (Jiangsu, China) in October 2017 for the further evaluation and diagnosis of the abnormal mass. After the hospitalization, the patient’s personal and medical records were found to be non-specific, the patient’s performance status was good, and the general physical examination did not reveal any abnormalities. Importantly, the chest enhanced CT revealed a 2.8 × 2.5 cm mass in the right posterior mediastinum (Fig. 1), and the abdominal CT ruled out any distant abnormal or metastasis lesions. Furthermore, the ultrasound gastroscope did not reveal any obvious abnormalities in the esophagus, while the bronchoscopy disclosed bloodstain in the posterior segment of the upper right lung, although the lavage, brush and sputum examinations were all negative. In addition, the patient complained of swelling in the right maxillary gingiva. The dental evaluation diagnosed the patient with periodontitis, and conservative treatment was given. Thereafter, the patient underwent the video-assisted thoracic surgery. During the procedure, the surgeon found that the posterior segment of the right upper lung lobe was adherent to the mediastinum, and that the tumor lesion was localized inside the lung. Thus, a Wedge resection of the right upper lung lobe was performed after separation of the adhesion. The frozen section diagnosis revealed a sarcomatoid carcinoma, resulting in the resection of the right upper lung lobe, together with the mediastinal lymph node dissections. The immunohistochemistry revealed that the sarcomatoid carcinoma was positive for vimentin, CKAE1/AE3 and Ki-67 (Fig. 2).

**Fig. 3:**
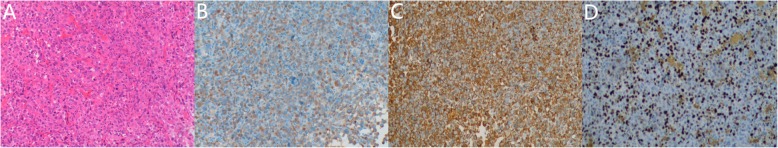
The histology and immunohistochemistry of the gingival tumor. **a** Histology. The hematoxylin and eosin (H&E) stained gingival tumor tissue shows that a number of heteromorphic cells infiltrated the subepithelial mesenchyme. These presented with a medium size and a relatively consistent morphology, indicating that these tumor cells had an abundant cytoplasm, red staining and obvious heteromorphism (× 100). **b** Immunohistochemistry. The data shows the partial positivity of the CKAE1/AE3 immunostaining in tumor cells. **c** Immunohistochemistry. The data shows the partial positivity of the vimentin immunostaining in tumor cells. **d** Immunohistochemistry. The data shows the partial positivity of the Ki-67 immunostaining in tumor cells

**Fig. 4:**
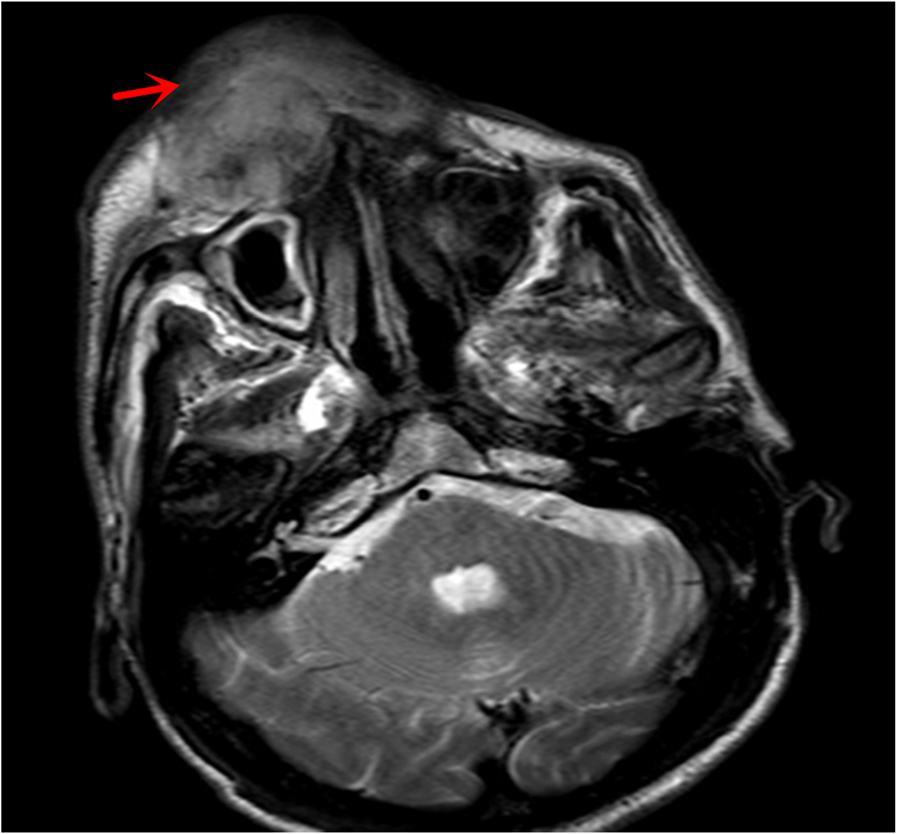
The nasopharyngeal MRI imaging during the chemotherapy. The MRI scan presents a 8 × 7 × 5.7 cm soft tissue mass localized at the right maxilla (red arrow), with an unclear boundary and local destruction of the maxilla and alveolar bone

During the patient’s recovery from surgery, the patient’s right maxillary gingival lesion gradually increased in size, and had bleeding. A tissue biopsy conducted by a dentist revealed a poorly differentiated tumor, and the immunostaining revealed that the tumor cells were positive for vimentin, CKAE1/AE3 and Ki-67 (Fig. 3). The biopsy histology disclosed that the tumor lesion was identical to lung cancer, indicating a metastatic PSC. Thus, the patient underwent radiotherapy, followed by chemotherapy. During the chemotherapy, the magnetic resonance imaging (MRI) scan localized a 8 × 7 × 5.7 cm soft tissue mass in the right maxilla with unclear boundary, and local destruction of the maxilla and alveolar bone (Fig. 4). Fifty days later, the patient stopped the chemotherapy due to poor systemic conditions, and was deceased as a result of multiple tumor metastases.

## Discussion

The World Health Organization classifies PSC as a group of poorly differentiated NSCLCs with sarcoma or sarcoma-like differentiation [1]. The PSC subcategories could be pleomorphic carcinoma, pure spindle-cell carcinoma, pure giant cell carcinoma, carcinosarcoma, and pulmonary blastoma. Recently, a study indicated that carcinosarcoma and pulmonary blastoma are histogenetically and morphologically distinct from the remaining tumors of this group, and its accurate subtyping and molecular and genetic classification could eventually lead to better therapy [4]. To date, the origin of the sarcoma component of PSC remains unclear, although previous studies have speculated that this is a clonal evolution [5, 6], which induced PSC to have the histological characteristics of both epidermal and mesenchymal tumors. Using immunohistochemistry, the present PSC case was confirmed with the positive staining of epithelial markers (CK and EMA) and a mesenchymal marker (Vimentin), while Ki67 positivity confirmed the aggressiveness of PSC in tumor cell proliferation.

According to literature, the male-to-female ratio of PSC prevalence is approximately 4:1, and the average age at PSC diagnosis is 60 years old, while most male patients have a history of moderate-to-heavy tobacco consumption [7]. The common clinical symptoms include cough, expectoration, and chest congestion and pain [8]. PSC diagnosis mainly depends on tumor tissue histology and the immunohistochemical staining of epithelial and mesenchymal markers. However, a CT scan may also provide certain imaging characteristics, such as the following: (1) The peripheral type is more than the central type, and tumor lesions are mostly localized in the upper lung lobe. (2) Most tumor lesions are large with a clear boundary, and a round or quasi-circular shape. However, due to tumor uneven growth, the lobulation of tumor lesions could occur, while the burrs are rare. (3) A plain CT scan may show tumor lesions as a dense soft tissue and large patchy necrosis occurring often inside the tumor lesion. Irregular thick-walled cavities or multiple small non-walled cavities within the necrosis may also occur due to the uneven tumor necrosis. (4) Most of tumor masses in an enhanced CT may reveal as mild-to-moderate rim annular enhancement or uneven patchy enhancement. (5) Peripleural masses are usually subpleural, and are prone to invasion to the pleura or chest wall, and these are often accompanied by local or distant metastasis. (6) Calcification within a tumor mass is rare. Thus, the CT initially identifies the tumor, and the histology and immunohistochemistry would subsequently confirm the PSC in clinic. In addition, compared to other histologic subtypes of NSCLC, PSC is more aggressive with poor prognosis. For example, the average survival duration of PSC patients is only 13.3 months, which is lower than other types of NSCLC due to tumor early metastasis [9]. Furthermore, PSC also frequently metastasizes to the esophagus, colon, rectum and kidneys, in addition to the common metastatic sites of NSCLC [10]. In clinic, the main method of treatment for PSC is surgery, while radiotherapy and chemotherapy are the auxiliary methods, although most patients suffer from the advanced stage of disease and lose surgery opportunity for cure. Furthermore, two-thirds of patients are not sensitive to conventional chemotherapy. Previous studies [11, 12] have revealed that R+ resection, pathologic TNM status, and vascular emboli are all independent prognostic factors. However, PSC prognosis remains presently poor, regardless of whether the PSC diagnosis is early or late, and whether tumor metastasis is present [13]. Thus, novel treatment options are needed to cure PSC patients, or improve the long-term survival.

A previous study [14] reported that high invasiveness of PSC is associated with tumor cell epithelial-mesenchymal transition (EMT), which could cause the carcinoma component of PSC to transform into a sarcoma component. The EMT can also induce the PSC to be highly invasive and potentially metastasize. Molecularly, EMT mutation and programmed death-ligand 1 (PD-L1) overexpression are associated with a particular propensity for sarcomatoid carcinoma [15]. Thus, EMT and PD-L1 may play a role in PSC cell epithelial-mesenchymal transformation, especially the epithelial-mesenchymal transformation to sarcomatoid carcinoma. Hence, targeting the EMT and PD-L1 may be a future research direction for the treatment of PSC patients.

## Conclusion

The present PSC case presented with variable clinical manifestations and a metastatic tumor in the gingiva. Thus, a differential diagnosis is needed to make the correct diagnosis for the primary and metastatic tumor, and CT and MRI scanning should be routinely performed for PSC patients.

## Data Availability

The authors declare that all data and materials of the article are available to all readers of our article.

## Abbreviations

CK: Cytokeratin
CT: Computed tomography
EMA: Epithelial membrane antigen
EMT: Epithelial-mesenchymal transition
MRI: Magnetic resonance imaging
NSCLC: Non-small cell lung cancer
PD-L1: Programmed death-ligand 1
PSC: Pulmonary sarcomatoid carcinoma

## References

[1] Travis WD, Brambilla E, Nicholson AG (2015). The 2015 World Health Organization classification of lung tumors: impact of genetic, clinical and radiologic advances since the 2004 classification. J Thorac Oncol.

[2] Yendamuri S, Caty L, Pine M (2012). Outcomes of sarcomatoid carcinoma of the lung: a surveillance, epidemiology, and end results database analysis. Surgery..

[3] Yoo SH, Han J, Kim TJ, Chung DH (2005). Expression of CD99 in pleomorphic carcinomas of the lung. J Korean Med Sci.

[4] Weissferdt A (2018). Pulmonary Sarcomatoid carcinomas: a review. Adv Anat Pathol.

[5] Thomas VT, Hinson S, Konduri K (2012). Epithelial-mesenchymal transition in pulmonary carcinosarcoma: case report and literature review. Ther Adv Med Oncol.

[6] Chang YL, Wu CT, Shih JY, Lee YC (2011). EGFR and p53 status of pulmonary pleomorphic carcinoma: implications for EGFR tyrosine kinase inhibitors therapy of an aggressive lung malignancy. Ann Surg Oncol.

[7] Martin LW, Correa AM, Ordonez NG (2007). Sarcomatoid carcinoma of the lung: a predictor of poor prognosis. Ann Thorac Surg.

[8] Franks TJ, Galvin JR (2010). Sarcomatoid carcinoma of the lung: histologic criteria and common lesions in the differential diagnosis. Arch Pathol Lab Med.

[9] Huang SY, Shen SJ, Li XY (2013). Pulmonary sarcomatoid carcinoma: a clinicopathologic study and prognostic analysis of 51 cases. World J Surg Oncol.

[10] Chang YL, Lee YC, Shih JY, Wu CT (2001). Pulmonary pleomorphic (spindle) cell carcinoma: peculiar clinicopathologic manifestations different from ordinary non-small cell carcinoma. Lung Cancer.

[11] Gu L, Xu Y, Chen Z, Pan Y, Lu S (2015). Clinical analysis of 95 cases of pulmonary sarcomatoid carcinoma. Biomed Pharmacother.

[12] Lococo F, Rapicetta C, Cardillo G (2017). Pathologic findings and long-term results after surgical treatment for pulmonary Sarcomatoid tumors: a multicenter analysis. Ann Thorac Surg.

[13] Yuki T, Sakuma T, Ohbayashi C (2007). Pleomorphic carcinoma of the lung: a surgical outcome. J Thorac Cardiovasc Surg.

[14] Pelosi G, Sonzogni A, De Pas T (2010). Review article: pulmonary sarcomatoid carcinomas: a practical overview. Int J Surg Pathol.

[15] Boland JM, Mansfield AS, Roden AC (2017). Pulmonary sarcomatoid carcinoma-a new hope. Ann Oncol.

